# Case Report: Anlotinib Combined With Sintilimab as Third-Line Treatment in a Metastatic Urothelial Bladder Carcinoma Patient With FGFR3 Mutation

**DOI:** 10.3389/fonc.2021.643413

**Published:** 2021-05-24

**Authors:** Jian-zhou Cao, Wei Wu, Jin-feng Pan, Hong-wei Wang, Jun-hui Jiang, Qi Ma

**Affiliations:** ^1^ Medical School, Ningbo University, Ningbo, China; ^2^ Comprehensive Urogenital Cancer Center, Ningbo First Hospital, The Affiliated Hospital of Ningbo University, Ningbo, China; ^3^ Department of Medical Oncology, Mingzhou Hospital, Ningbo, China; ^4^ Department of Pathology, People’s Hospital, The Affiliated Hospital of Ningbo University, Ningbo, China; ^5^ Department of Urology, Ningbo First Hospital, The Affiliated Hospital of Ningbo University, Ningbo, China; ^6^ Ningbo Clinical Research Center for Urological Disease, Ningbo, China; ^7^ Translational Research Laboratory for Urology, The Key Laboratory of Ningbo City, Ningbo First Hospital, The Affiliated Hospital of Ningbo University, Ningbo, China

**Keywords:** anlotinib, sintilimab, immunotherapy, targeted therapy, metastatic urothelial bladder carcinoma

## Abstract

We report on a case of metastatic urothelial bladder carcinoma (mUBC) treated with anlotinib combined with sintilimab. A 69-year-old male was diagnosed with non-muscle invasive bladder cancer (NMIBC). He received transurethral resection of bladder tumor (TURBT) and intravesical gemcitabine chemotherapy. After the patients’ cancer progressed to mUBC, cisplatin-based chemotherapy (gemcitabine combined with cisplatin, GC) was performed to this patient as first line therapy for four cycles. However, the disease progressed again within 6 months. Local radiotherapy was performed on the metastatic lesions, and after radiotherapy, the patient received anti-PD-1 antibody (sintilimab 200 mg, q3w)combined with Albumin-bound (Nab)-paclitaxel (100 mg, qw) as the second-line therapy, but the patient’s cancer was still observed to be progressing. Molecular characterization confirmed the presence of FGFR3 mutations in the patient. Anlotinib was recommended to this patient. After the patient was fully informed and he was aware of off-label use of the drug, then, Nab-paclitaxel was replaced by anlotinib (10 mg D1–14, q3w) and sintilimab infusions were maintained for every 3 weeks. Partial response (PR) was observed through imaging examinations and stable disease (SD) was observed for more than 11 months; the patient’s quality of life also improved. This case suggested that anlotinib combined with sintilimab may be a safe and effective choice in the treatment of mUBC in patients with FGFR3 mutations.

## Background

The prognosis of patients with mUBC is very poor if the disease progresses after platinum-based chemotherapy (1). The immune checkpoint inhibitors programmed cell death ligand-1 (PD-L1)/programmed death-1 (PD-1) are clinically active reagents for mUBC. Both of them are FDA-approved as second-line treatments (2). However, the objective response rate (ORR) was achieved in only 17% to 24% of these patients (3–7).

Fibroblast growth factor receptors (FGFRs) induce signaling through networks that regulate cell proliferation, survival, migration, and differentiation (8). FGFR3 is one of the most frequently mutated genes and is a promising target in urothelial carcinoma (UC) (9). FGFR3 is altered in 50% to 80% of low-grade and low-stage UC, particularly in the luminal I subtype. Conversely, FGFR3 mutations are less common (20%) in mUC (9, 10). FGFR inhibitors such as erdafitinib, when used to treat patients who had locally advanced or unresectable mUC with FGFR alterations, were shown to have an objective tumor response of 40% (11). The FDA has approved the use of erdafitinib for patients with locally advanced or mUC that has progressed during or after platinum-based chemotherapy and whose tumors have susceptible FGFR3 or FGFR2 genetic alterations (12).

Anlotinib is a novel multitarget tyrosine kinase inhibitor. It was originally designed to inhibit VEGFR2/3, FGFR1–4 with high affinity (13, 14). Clinical trials have indicated that anlotinib significantly prolongs the progression-free survival (PFS) of patients with non-small cell lung cancer (NSCLC) (13, 15, 16). medullary thyroid carcinoma (MTC) (13) and metastatic renal cell carcinoma (mRCC) (17). Sintilimab is an IgG4 monoclonal PD-1 antibody that was derived from humans, and it blocks the binding of PD-1 to PD-L1 or PD-L2 (18). It has been shown excellent clinical benefits in the treatment of relapsed or refractory Hodgkin’s lymphoma (19, 20) and NSCLC (21, 22).

Here we report on a 69-year-old male mUBC patient who had the FGFR3 mutations and was successfully treated with sintilimab combined with anlotinib as the third-line treatment, following the progression of cancer after first-line platinum-based chemotherapy and second-line Nab-paclitaxel plus sintilimab treatment.

## Case Presentation

**Figure 1:**
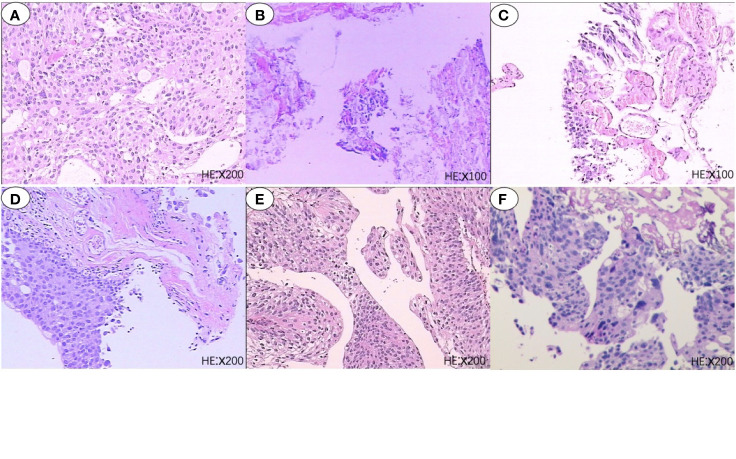
Six pathological reports of TURBT. **(A)** 2016-07-07, Papillary urothelial carcinoma, low-grade; **(B)** 2016-09-27, Necrotic tissue; **(C)** 2017-06-15, Chronic inflammation of the bladder mucosa with atypical hyperlasia; **(D) **2017-12-14, Chronic inflammation of the bladder mucosa with atypical hyperlasia; **(E)** 2018-05-10, Papillary urothelial carcinoma, low-grade; **(F)** 2018-11-23, Papillary urothelial carcinoma, high-grade.

**Figure 2:**
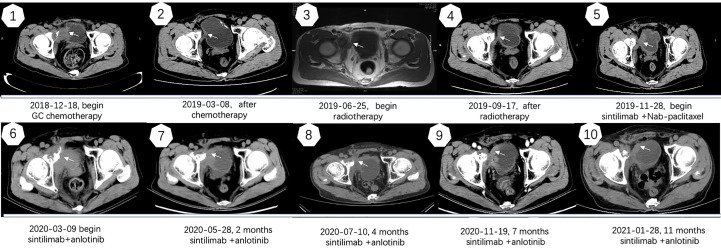
Imaging manifestations of treatment.

A 67-year-old man with no family and psychosocial history presented with hematuria in 2016 and was diagnosed with bladder carcinoma using cystoscopy. TURBT pathology indicated that the patient has stage-TaG1 UBC (**Figure 1A**). Single-dose intravesical gemcitabine chemotherapy within 24 hours after receiving TURBT was recommended for the patient. However, 2 months after TURBT was performed, cystoscopy revealed bladder carcinoma recurrence. Between July 2016 and May 2018, TURBT was repeated five times due to cancer recurrence and progression (**Figures 1B–F**). After the fifth TURBT was performed, abdominal computed tomography (CT) (**Figure 2**-1) revealed bladder carcinoma recurrence and pelvic metastasis. Further evaluation of the pelvis using magnetic resonance imaging (MRI) suggested multiple metastatic foci of the pelvic muscle 、bone and lymph nodes. Pelvic bone metastases were also found using emission computed tomography (ECT). The patient was diagnosed as mUBC on December 13, 2018. At that time, the ECOG score of this patient was 0 and the glomerular filtration rate (GFR) was 69 ml/min. He was treated with first-line cisplatin-based chemotherapy (gemcitabine combined with cisplatin, GC) for four cycles. The disease obtained an objective response and was stable for 6 months, after which imaging suggested that the disease progressed again. As the patient responded to GC chemotherapy previously, the patient received another two cycles of GC chemotherapy. Unexpectedly, the patient experienced severe pain and could not tolerate the toxicity of chemotherapy. Next, the patient received radiotherapy to the metastatic lesions from July to August 2019, with a total dose of 55 Gy, 2.2 Gy daily, up to 25 fractions to control pain and improve local cancer control. Then, anti-PD-1 antibody (sintilimab 200 mg, q3w) combined with Nab-paclitaxel (100mg, qw) was given to this patient as the second-line therapy on November 28, 2019. Unfortunately, after four cycles treatment, the patient’s abdominal CT (**Figure 2**–6) showed that the treatment had failed and the patients’ condition continued to deteriorate. He experienced severe bladder irritation due to tumor progression and radiation cystitis, the patient required 120 mg of Morphine Sulfate sustained-release tablets to control local pain.

**Figure 3:**
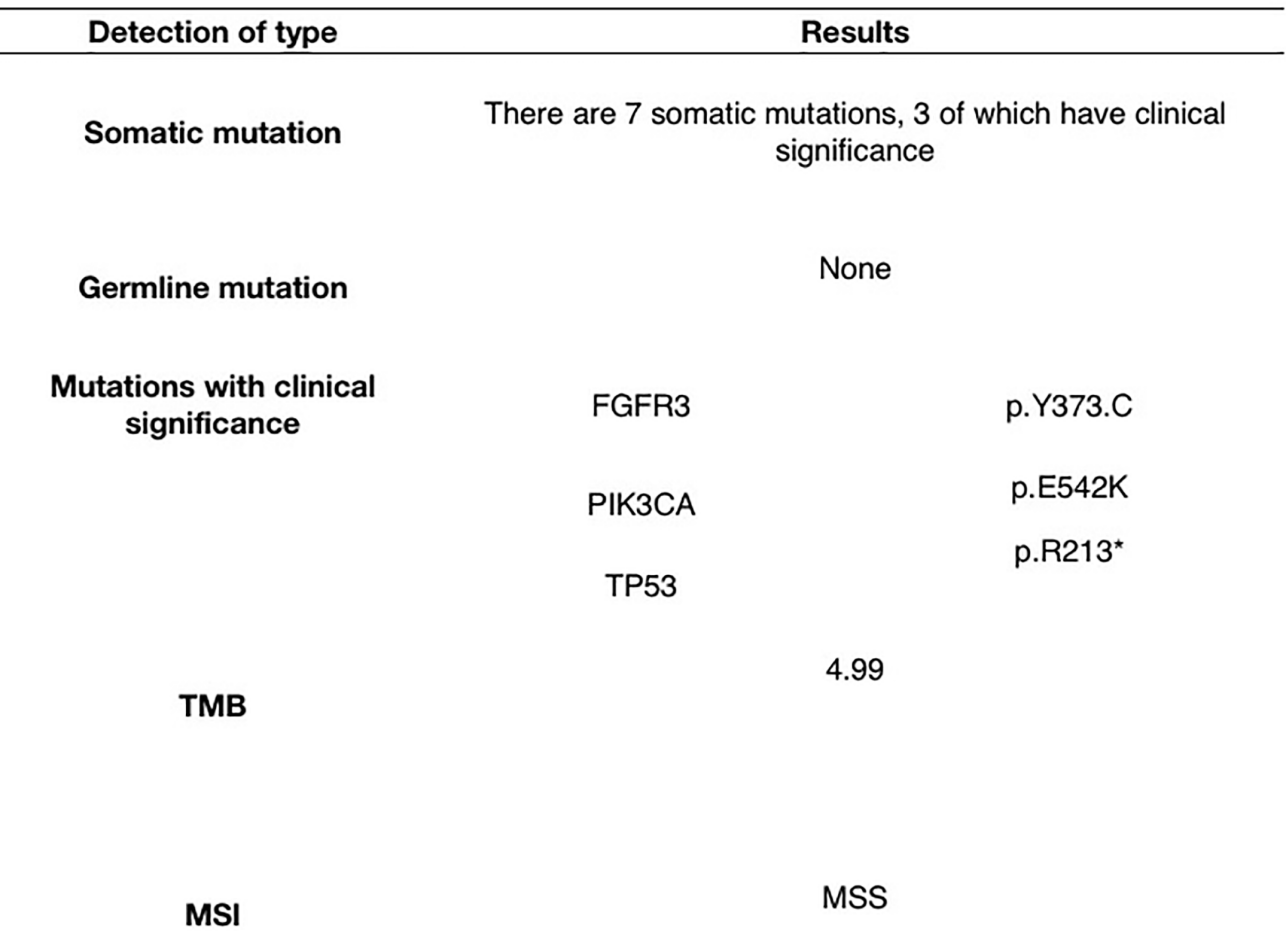
The molecular characterization.

To provide further treatment to this patient, we performed genetic sequencing and molecular characterization confirmed the presence of FGFR3, PIK3CA, and TP53 mutations in the patient (**Figure 3**). Both erdafitinib and rogaratinib have been tested in clinical trial for FGFR mutated mUBC (23), however, both drugs are not affordable in China. We encouraged the patient to enrollment a clinical trial (NCT03390504) sponsored by Johnson & Johnson (24), which treated mUBC patients with FGFR mutation by erdafitinib. However, the patient did not have strong intention to enroll into this trial.

**Figure 4:**
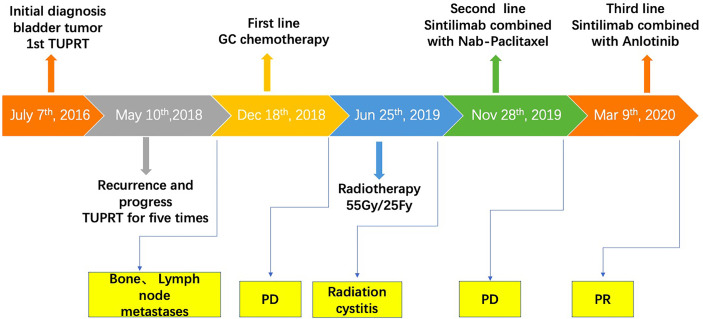
The timeline of treatment.

Thus, after careful consideration, anlotinib, a multitarget tyrosine kinase inhibitor designed to inhibit VEGFR2/3, FGFR1–4 with high affinity, was recommended to this patient. The patient was fully informed and he was aware of off-label use of the drug. With the patient’s consent, Nab-paclitaxel was changed to anlotinib (10 mg D1-14 q3w) on March 9, 2020, and the infusion of sintilimab was maintained. After treatment with sintilimab combined with anlotinib, the patient’s symptoms improved within 2 weeks and the dose of Morphine Sulfate sustained-release tablets was decreased and eventually stopped. After three cycles, the disease was evaluated by abdominal CT (**Figure 2**) and PR was observed. Currently, the cancer has been stable for over 11 months and the patient is still being followed up. Except for a mild Hand-foot syndrome, no serious adverse events(AE) occurred while receiving sintilimab combined with anlotinib. The patient has not significant decreased in quality of life during treatment and he is quite satisfied with the outcome. The timeline of the patients’ treatment is described in **Figure 4**.

## Discussion

Platinum-based chemotherapy can prolong overall survival (OS) in patients with mUBC, but cancer progression is almost inevitable (25). Nab-paclitaxel as a second-line treatment has demonstrated some positive preliminary activity in patients with mUBC (26). Yoo-Joung Ko reported on a single-group phase II trial which investigated the activity of Nab-paclitaxel 260 mg/m^2^ every 3 weeks as a second-line therapy for mUBC (26, 27). The results suggested that Nab-paclitaxel was well tolerated, but the clinical effect of Nab-paclitaxel monotherapy was limited (27). Recently, PD-1/PD-L1 inhibitors have been demonstrated to be relatively safe and have shown positive clinical activity in patients with mUBC (2, 6, 28), however, the ORR of single PD1/PD-L1 treatment was only 17% to 24% (3–7). To further improve PD1/PD-L1 treatment efficacy, PD-1 was combined with Nab-paclitaxel as a second-line therapy for mUBC. The open-label, single-arm, phase II PEANUT study found that the Pembrolizumab and Nab-paclitaxel salvage therapy for platinum-treated failed mUBC had a favorable safety profile, the PFS was 5 months, and the clinical ORR was 44.4% (29). Thus, when the patient was found that the disease progressed quickly after first line GC therapy, we tried to combine Nab-paclitaxel and PD-1/PD-L1 to control the disease.

Although five kinds of PD-1/PD-L1 are FDA approved for second-line treatments of mUBC (12), only Pembrolizumab and Nivolumab were available in China when the patient decided to receive PD-1/PD-L1 therapy. However, both Pembrolizumab and Nivolumab did not get formal permission for indication of mUBC treatment in China. Moreover, both drugs were too expensive to use. Thus, after careful discussion with the patient, we decided to choose another cheaper PD-1 antibody to substitute Pembrolizumab or Nivolumab.

According to the pre-clinical data, sintilimab binds to human PD-1 with a greater affinity than nivolumab and pembrolizumab (18). The high binding affinity and unique PD-1 epitopes bound by sintilimab might be responsible for its superior clinical effectiveness (18). Sintilimab has shown excellent clinical benefits in the treatment of relapsed or refractory Hodgkin’s lymphoma (19, 20) and NSCLC (21, 22). Theoretically, sintilimab combined with Nab-paclitaxel may produce favorable results for this patient, however, in this case, the combination was unsuccessful.

Genetic testing confirmed the presence of FGFR3, PIK3CA, and TP53 mutations in the patient. FGFR3 mutations are associated with a lower response to platinum-based chemotherapy and a shorter recurrence time in patients with mUBC (30, 31), which was consistent with this patient who received six cycles of platinum-based chemotherapy. Erdafitinib, a pan-FGFR inhibitor, has been granted accelerated approval by the FDA for platinum-pretreated mUBC with susceptible to FGFR3 or FGFR2 genetic alterations (32), but Erdafitinib is unaffordable in China.

Anlotinib was originally designed to inhibit VEGFR2/3, FGFR1–4 with high affinity (13, 14). Anlotinib also suppresses the activity of PDGFRα/β, c-Kit, Ret, Aurora-B, c-FMS, proving that it has broad inhibitory effects on tumor proliferation, vasculature, and tumor microenvironment (13, 14). In clinical trials, anlotinib showed broad antitumor activity against a variety of tumors. In advanced refractory solid tumors, anlotinib displayed manageable toxicity, and broad-spectrum antitumor potential (13). In locally advanced or metastatic MTC, 56.9% of patients experienced a PR after anlotinib treatment. PFS rate at 48 weeks was 85.5% (33). In a phase II trial on 166 patients with refractory metastatic soft-tissue sarcoma, the median progression-free survival and OS were 5.6 and 12 months, respectively (14). Similarly, in advanced NSCLC, Anlotinib appeared to lead to prolonged OS and PFS. OS was significantly longer in the anlotinib group than the placebo group (9.6 months *vs* 6.3 months) (16). Furthermore, anlotinib demonstrated that it had a better prognosis compared to sunitinib as the first-line treatment for patients with mRCC in a randomized phase II trial (17). In this trial, Anlotinib’s safety profile was excellent, especially in terms of hematological toxicities (17).

In addition to anti-tumor efficacy, anlotinib has the potential to modulate the tumor microenvironment and improve immunotherapy. A lung cancer mouse model showed that anlotinib could increase infiltration of innate immune cells such as natural killer (NK) cells and antigen-presenting cells (APC) into tumor microenvironment (15). Subsequently, when combined with PD-1/PD-L1 blockade, anlotinib provided significantly synergistic therapeutic benefits (15). A retrospective study further demonstrated the efficacy and safety of anlotinib with immunotherapy in advanced NSCLS as a third-line therapy (34). Based on these studies, we attempted to treat this patient by anlotinib combined with sintilimab, and favorable results were obtained.

To our knowledge, this is the first report that used anlotinib combined with sintilimab as the third-line treatment in an mUBC patient with FGFR3 mutation, who obtained a PR and was stable for more than 11 months. This case indicates that mUBC patients with FGFR3 mutations whose disease progresses after platinum-based chemotherapy may be able to use anlotinib combined with sintilimab as a new potential treatment choice, but we have only one case and further studies should be conducted to evaluate the efficacy and safety of this combination.

## Data Availability Statement

The original contributions presented in the study are included in the article/supplementary material. Further inquiries can be directed to the corresponding authors.

## Ethics Statement

The studies involving human participants were reviewed and approved by the ethical review committee of Ningbo First Hospital. The patients/participants provided their written informed consent to participate in this study.

## Author Contributions

Conception/design: QM. Provision of study materials or patients: QM, WW, and J-HJ. Collection and/or assembly of data: QM, J-ZC, J-FP, WW, and H-WW. Data analysis and interpretation: QM and J-ZC. Manuscript writing: J-ZC and QM. Final approval of manuscript: All authors. All authors contributed to the article and approved the submitted version.

## Funding

This study was supported by Zhejiang Natural Science Fund (grant no. LY20H050002 to QM, grant no. LY18H05000 to J-HJ), Ningbo Natural Science Fund (grant no. 2018A610297 to QM), Ningbo Social Development Fund (grant no. 202002N3192 to QM), and the Fund of Ningbo Clinical Research Center for Urological Disease (2019A21001).

## Conflict of Interest

The authors declare that the research was conducted in the absence of any commercial or financial relationships that could be construed as a potential conflict of interest.
